# TIM‐4 interference in Kupffer cells against CCL4‐induced liver fibrosis by mediating Akt1/Mitophagy signalling pathway

**DOI:** 10.1111/cpr.12731

**Published:** 2019-11-22

**Authors:** Hao Wu, Guoyong Chen, Jingyuan Wang, Minghua Deng, Fangchao Yuan, Jianping Gong

**Affiliations:** ^1^ Department of Hepatobiliary Surgery The Second Affiliated Hospital of Chongqing Medical University Chongqing China; ^2^ Department of Hepatobiliary and pancreatic surgery Henan Provincial People’s Hospital People’s Hospital of Zhengzhou University People’s Hospital of Henan University Zhengzhou China

**Keywords:** Kupffer cells, liver fibrosis, liver transplantation, mitophagy, T‐cell immunoglobulin domain and mucin domain‐4

## Abstract

**Objectives:**

T‐cell immunoglobulin domain and mucin domain‐4 (TIM‐4) is selectively expressed on antigen‐presenting cells (APCs) and modulates various immune responses. However, the role of TIM‐4 expressed by Kupffer cells (KCs) in liver fibrosis remains unclear. The present study aimed to explore whether and how TIM‐4 expressed by KCs is involved in liver fibrosis.

**Materials and Methods:**

Mice chronic liver fibrosis models were established and divided into the olive‐induced control group, CCL4‐induced control group, olive‐induced TIM‐4 interference group and CCL4‐induced TIM‐4 interference group. Different techniques were used to monitor the fibrotic effects of TIM‐4, including histopathological assays, Western blotting, ELISA and transmission electron microscopy. Additionally, mice liver transplant models were established to determine the fibrotic effects of TIM‐4 on fibrosis after liver transplantation (LT).

**Results:**

We found that the induction of liver fibrosis by CCL4 was associated with TIM‐4 expression in KCs. TIM‐4 interference essentially contributed to liver fibrosis resolution. KCs from the TIM‐4 interference group had decreased levels of pro‐fibrotic markers, reduced TGF‐β1 secretion and inhibited hepatic stellate cell (HSC) differentiation into myofibroblast‐like cells. In addition, we used GdCl3 to verify that KCs are the primary source of TGF‐β1 during fibrosis progression. Moreover, KCs from CCL4‐induced mice showed increased ROS production, mitophagy activation and TGF‐β1 secretion. However, TIM‐4 interference in the KCs inhibited Akt1‐mediated ROS production, resulting in the suppression of PINK1, Parkin and LC3‐II/I activation and the reduction of TGF‐β1 secretion during liver fibrosis. Additionally, TIM‐4 interference potentially attenuated development of fibrosis after LT.

**Conclusions:**

Our findings revealed the underlying mechanisms of TIM‐4 interference in KCs to mitigate liver fibrosis.

## 1 INTRODUCTION

Liver fibrosis is the consequence of a sustained wound healing response to chronic liver injury, which is characterized by the excessive deposition of extracellular matrix (ECM).^1^ Notably, liver fibrosis substantially accelerates the risk of cirrhosis and hepatocellular carcinoma and has resulted in a major public health problem worldwide.^2^, ^3^ Many contributing factors are implicated in the aetiology of liver fibrosis. For example, viral infection, especially hepatitis B virus, is the major contributor to liver fibrosis in China.^4^, ^5^ In Western countries, the prevailing causes of fibrosis and cirrhosis include chronic alcohol consumption and non‐alcoholic fatty liver disease associated with obesity and type 2 diabetes.^6^ In addition, drug‐induced toxicity, immune‐mediated liver diseases, metabolic disorders and cholestasis are also associated with liver fibrosis occurrence and progression.^2^, ^7^ Liver transplantation (LT) is considered to be the only option for end‐stage liver disease. However, due to the shortage of liver donors, there is an urgent need to develop therapeutics with well‐elucidated molecular mechanisms for the treatment of liver fibrosis.

Previous studies have shown that activated hepatic stellate cells (HSCs) are responsible for ECM deposition during the progress of liver fibrosis.^8^ The fibrogenic process is initiated by reiterative hepatocyte or biliary cell death, inflammation and oxidative stress, leading to the differentiation of HSCs into myofibroblast‐like cells at the site of injury.^9^ Recent studies have underscored that sustained inflammation originating from resident immune cells is central regulators of liver fibrosis and contribute to its resolution.^10^ In the occurrence and progression of liver fibrosis, the inflammatory response is a necessary process, and macrophages are the primary cells in the inflammatory response. Particularly, it is well established that hepatic macrophages promote the activation and survival of hepatic stellate cells and function in the initiation and progression of liver fibrosis.^11^, ^12^ In addition, the inhibition of monoacylglycerol lipase in hepatic macrophages promotes fibrosis resolution during chronic liver injury.^13^ Collectively, these data indicate that targeting hepatic macrophages to control fibrosis progression may be a promising therapeutic strategy to reverse liver fibrosis.

The T‐cell immunoglobulin domain and mucin domain (TIM) gene family was identified and cloned in 2001 using asthma mice models.^14^ The TIM gene family consists of 8 members in the mouse (TIM‐1‐8) and human chromosome (TIM‐1, 3 and 4), and all of their amino acid sequences are highly homologous. Previous studies have demonstrated that TIM members are implicated in immune responses, including autoimmunity, transplant tolerance, allergic diseases and the response to viral infections. TIM‐1 is only expressed in primary T cells as a co‐stimulatory signal for T‐cell activation^15^; TIM‐2 is selectively expressed in Th2 cells and negatively regulates the proliferation and differentiation of Th2 cells^16^; TIM‐3 is expressed in activated Th1 and Th17 cells, binding to galectin‐9 to induce Th1 and Th17 cell apoptosis.^17^ Unlike the aforementioned TIM molecules, TIM‐4, identified as a phosphatidylserine (PS) receptor mediating the uptake of apoptotic cells, is the only non‐T‐cell TIM protein and is selectively expressed on the surface of antigen‐presenting cells (APCs), particularly in macrophages. The limitation of this expression suggests that TIM‐4 may play an important role in the function of APCs.^18^ Upon stimulation of mouse liver mononuclear cells with LPS in vitro, TIM‐4 expression was shown to be upregulated. In addition, spleen CD11b+ cells highly expressed TIM‐4 after LPS injection in vivo, that is TIM‐4 expression was upregulated after macrophage activation.^19^ Moreover, TIM‐4 can regulate the adaptive immune response by interacting with its natural ligand TIM‐1 to induce naive CD4+ T‐cell activation and differentiation.^20^ Kupffer cells (KCs) are the largest group of APCs, accounting for 80%‐90% of all monocyte‐macrophage cell groups.^21^ It has been indicated that blocking TIM‐4 expression in mice mitigates toll‐like receptor (TLR)‐4–mediated inflammation in liver ischaemia‐reperfusion injury.^22^ Additionally, we have previously reported that activated KCs highly express TIM‐4, and the TIM‐4 blockade of KCs attenuates acute rejection following liver transplantation in mice.^23^ However, the current understanding regarding the action of TIM‐4 is limited, and whether TIM‐4 is involved in liver fibrosis remains unaddressed. Our data have shown that TIM‐4 regulation in KCs is associated with Akt1‐mediated mitophagy, which is required for KC‐derived TGF‐β1 expression during liver fibrosis.

## 2 METHODS

### 2.1 Experimental animals

Eighty adult male C57 mice weighing 25‐30 g were purchased from the Animal Experimental Center of Chongqing Medical University (Chongqing, China). All mice were housed in cages in a room with a controlled temperature of 23℃ and 60% humidity under a 12‐h light/12‐h dark cycle. The mice had ad libitum access to food and water. All protocols were approved by the Animal Care and Use Committee of Chongqing Medical University (Chongqing, China), and no mice were sacrificed unexpectedly during all experiments. The mice were randomly categorized into four main groups: the olive‐induced control group (group I, n = 15), CCl4‐induced control group (group II, n = 15), olive‐induced TIM‐4 interference group (group III, n = 15) and CCL4‐induced TIM‐4 interference group (group IV, n = 15). Chronic liver fibrosis was induced in the mice of groups II and IV by intraperitoneal injection of CCl4 (1 µL of 10% CCL4/g, twice per week, for six weeks). Mice in groups I and III were injected with commensurable olive (twice per week, for six weeks). Meanwhile, during the induction period of chronic liver fibrosis, the mice of group III and IV were administered TIM‐4 siRNA (2 mg/kg, once per week) by tail vein injection, and the group I and II mice were injected with commensurable control vehicle.

Orthotopic liver transplantation models were established with an improved Kamada's two‐cuff method, as previously described.^24^ The sham group (n = 5) did not receive a transplant but still underwent abdominal cutting and vascular exposure around the liver and were administered TIM‐4 interference after surgery; the liver transplantation (LT) group (n = 5) received a transplant operation and were administered TIM‐4 interference after surgery. Both groups were injected with commensurable vehicle as control (n = 5).

### 2.2 KC and HSC isolation

Mouse livers were perfused using the in situ method described by Li *et al*,^25^ and the KCs were isolated and cultured in complete medium. The non‐adherent cells were removed, and the KCs were cultured in vitro. The cells or supernatants were collected for further analysis. Mouse livers were perfused using the in situ method described by Weiskirchen *et al* 26 HSCs were isolated and cultured in complete medium. Then, HSCs were cultured in the transwells, and KCs were co‐cultured with HSCs (at a ratio of 2:1) in the relevant well plates for 24 hours. Cells or supernatants were collected for further analysis.

### 2.3 Small interfering RNA (SIRNA)

TIM‐4 siRNA, Parkin siRNA and TGF‐β1 siRNA (Santa Cruz Biotechnology, CA, USA) were premixed with mannose‐conjugated polymers (greater than 175 nm in diameter, Polyplus‐transfection, USA) at a ratio specified by the manufacture, and Parkin siRNA or TGF‐β1 siRNA was administered by tail vein injection (2 mg/kg) as described before (n = 5 mice/ group).

### 2.4 Histological evaluation

Mouse liver tissues were fixed with 10% neutral formaldehyde solution, dehydrated with gradient ethanol and further embedded in paraffin. Paraffin‐embedded liver samples were sectioned (thickness approximately 3 μm) and stained with haematoxylin‐eosin (HE), Masson's trichrome and Sirius red according to manufacture protocols. The sections were sealed with neutral gum and observed under a light microscope.

### 2.5 Hydroxyproline determination

Liver tissues were dried to a stable weight and acid hydrolysed with 6 N HCl for 24 hours at 1120°C. The hydroxyproline concentration was normalized to the dry weight of the liver, as previously described.^27^

### 2.6 Immunoassays

Total protein was prepared from liver tissues and cells using a lysis buffer kit according to the manufacturer's instructions, and the protein concentration was determined using a BCA protein assay kit (Sangon Biotech, Shanghai, China). Immunohistochemistry was used to determine the expression of TIM‐4 (cat. no. GTX14149; 1:100; GeneTex Inc), HGF (cat. no. ab83760; 1:100; Abcam Inc), α‐SMA (cat. no. ab32575; 1:100; Abcam Inc) and collagen 1α (cat. no. ab34710; 1:100; Abcam Inc) in liver tissue according to the manufacturer's instructions. Western blotting assays were performed using the total protein extracted from liver tissues and cells. The total protein was separated on an SDS‐PAGE gel. The proteins were then electrotransferred onto PVDF membranes, which were blocked using 5% non‐fat milk dissolved in TBST. The membranes were incubated overnight at 4℃ with primary antibodies including TIM‐4 (cat. no. GTX14149; 1:1000; GeneTex Inc), α‐SMA (cat. no. ab32575; 1:1000; Abcam Inc), TGF‐β1 (cat. no. ab92486; 1:1000; Abcam Inc), PINK1 (cat. no. ab23707; 1:1000; Abcam Inc), Parkin (cat. no. ab77924; 1:100; Abcam Inc), p62 (cat. no. ab56416; 1:1000; Abcam Inc), LC3I/II (cat. no. ab51520; 1:1000; Abcam Inc), p‐Akt1 (cat. no. ab81283; 1:1000; Abcam Inc), Akt1 (cat. no. ab182729; 1:1000; Abcam Inc), cleaved caspase‐3 (cat. no. ab49822; 1:1000; Abcam Inc) and β‐actin (cat. no. 14395‐1‐AP; 1:1000; Proteintech Inc). They were then blotted with species‐matched secondary antibodies. The protein bands were visualized using a Bio‐Rad ChemiDocTM XRS system (Hercules, CA). All images were analysed using NIH ImageJ software. In addition, the levels of IL‐1β, TNF‐α, IL‐10, TGF‐β1, IL‐4 and IL‐13 were measured in liver tissue lysates and cell supernatants using an ELISA kit according to the manufacture's protocols (eBioscience, San Diego, CA, USA).

### 2.7 Immunofluorescence staining

Liver tissues were fixed in 4% paraformaldehyde and then dehydrated with gradient ethanol and xylene. The sections were then incubated in 1% triton X‐100 for 15 minutes. After eliminating endogenous peroxidase activity with 3% hydrogen peroxide for 15 minutes, the sections were incubated with primary anti‐F4/80 (cat. no. ab6640; 1:100; Abcam Inc) and anti‐TIM‐4 (cat. no. GTX14149; 1:1000; GeneTex Inc) at 4℃ overnight. Next, the sections were washed with PBS and incubated with species‐matched secondary antibodies for 30 minutes at room temperature. The sections were then washed with PBS and sealed with FluorFluoromount‐G™ slide mounting medium (Southern Biotech, Birmingham, AL, USA). Images were acquired using a fluorescence microscope and were analysed using an Image Analysis system, version 11.0 (Chang Heng Rong Technology, Beijing, China).

### 2.8 Detection of liver function

Blood samples were collected from the abdominal aorta of liver transplant models, and the alanine aminotransferase (ALT) and aspartate aminotransferase (AST) levels in the serum were detected by an automated chemistry analyser (Beckman Coulter, CA, USA).

### 2.9 Quantitative RT‐PCR

Total RNA was extracted from liver tissues and cells using TRIzol reagent (Takara, Otsu, Japan) and was reverse transcribed into cDNA using a PrimeScript™ 1st Strand cDNA synthesis kit (Takara, Otsu, Japan). qPCR assays were performed using SYBR Premix Ex Taq (Takara Bio Inc) and a cDNA template on an Applied Biosystems 7500 Real‐time PCR system (Applied Biosystems; Thermo Fisher Scientific Inc). The results were normalized against β‐actin expression, and RNA enrichments were calculated using the 2^‐ΔΔcq^ method.^28^

### 2.10 Apoptosis detection and flow cytometry analysis

Liver tissues were determined a using TUNEL kit (cat. no. 11684817910; Roche Inc, Switzerland) following method described by Kitamoto *et al* 29 KCs were harvested and washed twice with pre‐cooled PBS, then resuspended in binding buffer and stained with annexin V and PI (cat. no. A026; GeneCopoeia), followed by lucifugal incubation for 15 minutes at 37°C. Flow cytometric data were acquired using a FACScalibur and analysed using CellQuest version 5.1 software (BB Biosciences, NJ, USA).

### 2.11 Transmission electron microscopy

KCs were fixed with 2.5% paraformaldehyde and 2.5% glutaraldehyde in Sorenson's phosphate buffer at pH 7.4. The cells were then sectioned at 70 ~ 80 nm, and sections were placed on copper mesh grids. The sections were stained with uranyl acetate and lead citrate for contrast and examined with a transmission electron microscope (Jeol, 100 CXII).

### 2.12 Determination of ROS generation

The production of H2O2 in KCs was detected as previously described.^30^ Mitochondria in KCs were stained with 50 μmol/L MitoTracker green (Molecular Probes), and ROS colocalization was stained with 5 Mm dihydroethidium derivative (MitoSOX) (Molecular Probes). Images were acquired using a Zeiss LSM 510 confocal microscope (Zeiss AG, Thornwood, NY, USA).

### 2.13 Statistical analysis

All values are expressed as the mean ± SD. The data were analysed by unpaired two‐tailed Student's t test or one‐way analysis of variance with a post hoc test. SPSS 22.0 software was applied for all statistical analyses. A *P* value less than .05 was required for results to be considered statistically significant.

## 3 RESULTS

### 3.1 TIM‐4 expression of KCs is increased in liver fibrosis

We successfully established CCL‐4‐induced liver fibrosis models and found that there was extensive destruction of liver structure, along with abnormal collagen deposition, but olive‐induced models have normal liver architecture compared with the NC group (Figure S1A). The histological findings were verified biochemically by hydroxyproline assay, and there was no statistical difference between olive group and NC group (Figure S1B). Thus, we used olive‐induced models as control for further study.

**Figure 1:**
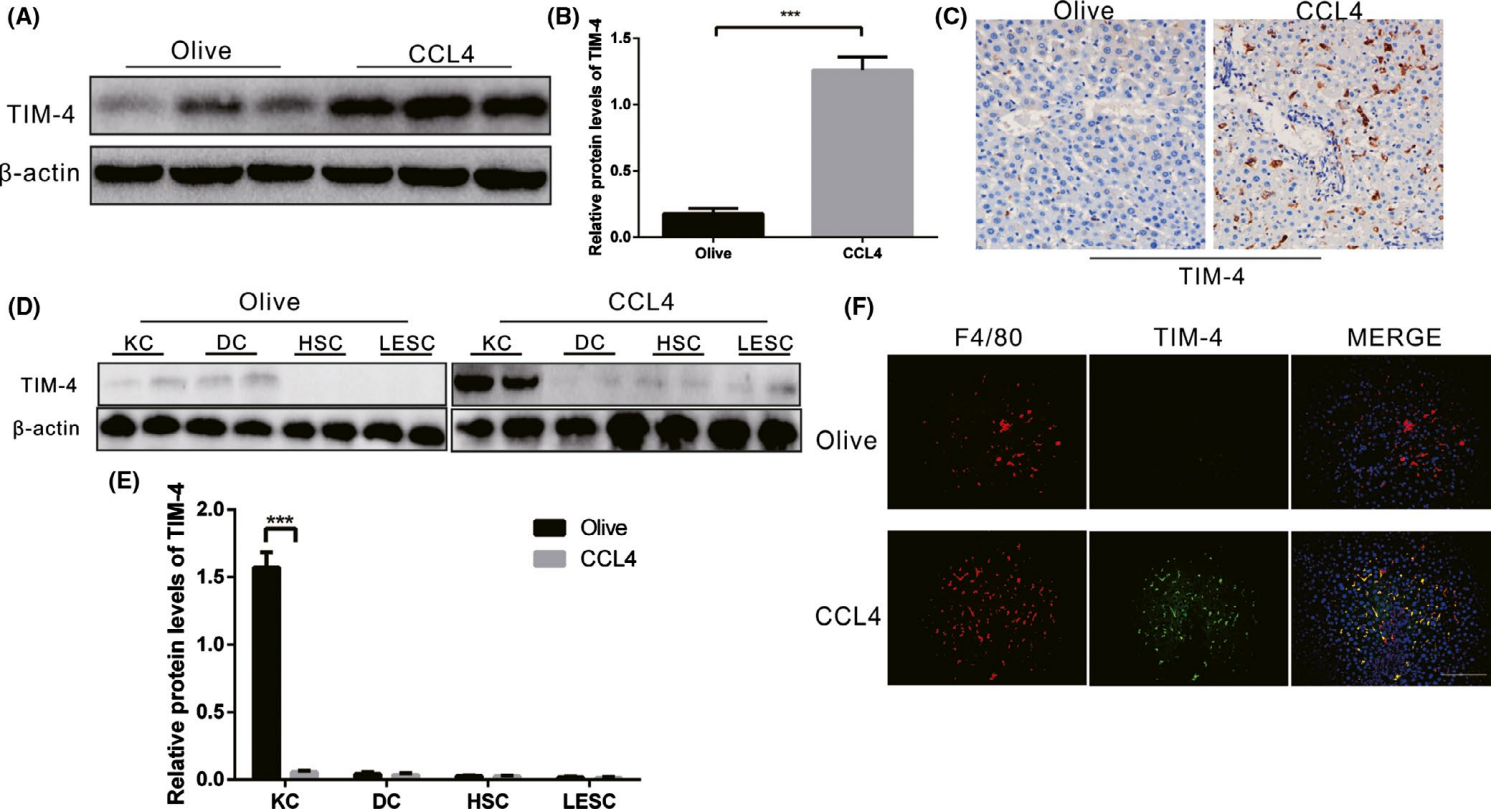
TIM‐4 in KCs is increased during liver fibrosis. A, B, Immunoblot and quantitative analysis of TIM‐4 expression in olive‐induced and CCL4‐induced liver (n = 3 mice/ group). C, The expression levels of TIM‐4 in olive‐induced and CCL4‐induced liver were assessed using immunohistochemistry (n = 3 mice/ group, magnification, ×400). D, Kupffer cells (KCs), dendritic cells (DCs), hepatic stellate cells (HSCs) and liver sinusoidal endothelial cells (LESCs) were isolated from olive‐induced and CCL4‐induced mice, and the (E) expression levels of TIM‐4 were assessed with immunoblot (n = 3 mice/ group). F, F4/80 (red) and TIM‐4 (green) expression in olive‐induced and CCL4‐induced liver tissues were detected by immunofluorescence (n = 3 mice/ group, magnification ×400, Scale bars: 50 μm). ***P < .0001. Values are the mean ± SD of a minimum of three independent experiments

To investigate whether TIM‐4 expression is influenced by liver fibrosis, we measured the expression of hepatic TIM‐4 in CCL4‐induced liver injury mice. CCL4‐induced mice had a marked increase in TIM‐4 expression compared to the olive group (Figure 1A,B). Livers from CCL4‐induced mice showed high, positive TIM‐4 expression, whereas livers from olive‐induced mice showed a negative result (Figure 1C). Then, the whole macrophages extracted from olive‐ and CCL4‐induced models’ livers were identified with F4/80 and CD11b by flow cytometry. The number of F4/80+ CD11b‐ cells (KCs) was predominant (>90%) in olive‐ and CCL4‐induced livers, whereas only a small percentage of F4/80 + CD11b+ (peripheral macrophages) were observed in olive‐ and CCL4‐induced livers (Figure S2A,B). So, we used KCs as the main research cells for further study. We then assessed which types of liver parenchyma cells were primarily expressing TIM‐4. KCs, dendritic cells (DCs), hepatic stellate cells (HSCs) and liver sinusoidal endothelial cells (LESCs) were isolated from CCL4‐induced and olive‐induced mice, but only KCs isolated from the livers of CCL4‐induced mice had dramatically increased TIM‐4 expression, which was 12‐fold greater than that in the olive mice (Figure 1D,E). The KCs from liver tissue were labelled with F4/80 (red). The expression levels of TIM‐4 (green label) in the CCL4‐induced liver tissue were elevated and colocalized with the F4/80 (red) fluorescence (Figure 1F). Colocalization was not found in the olive‐induced liver tissues. These findings suggest that TIM‐4 was mainly expressed in KCs after CCL4‐induced liver fibrosis and therefore may be associated with liver fibrosis.

### 3.2 TIM‐4 interference contributes to liver fibrosis resolution

**Figure 2:**
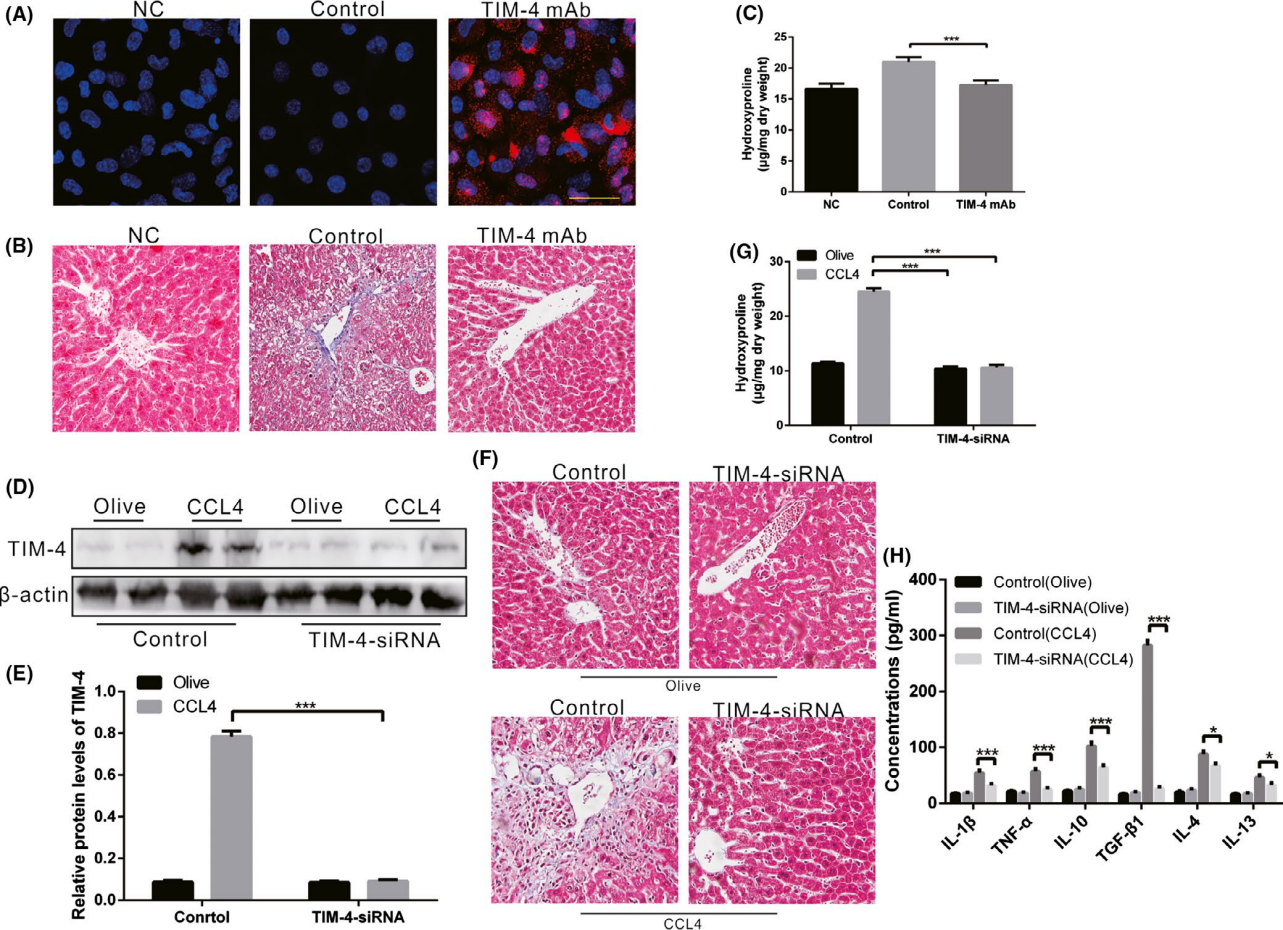
TIM‐4 in KCs is associated with liver fibrosis. A, KCs were isolated from mice with TIM‐4 mAb (red fluorescence label) treatment or control mAb treatment and then examined using laser confocal microscopy to identify the successful blocking of TIM‐4 function (n = 3 mice/ group, Scale bars: 50 μm). Negative control (NC) as negative control without any treatment. B, Liver tissues from TIM‐4 mAb and control mice were processed for Masson's trichrome staining (n = 3 mice/ group, magnification, ×400). NC as negative control without any treatment. C, Hydroxyproline of livers removed from olive‐induced and CCL4‐induced mice with or without TIM‐4 mAb treatment (n = 3 mice/ group). D, E, Mannose‐conjugated polymers were used to deliver TIM‐4 siRNA or its scrambled control siRNA in vivo, and the expression levels of TIM‐4 in olive‐induced and CCL4‐induced liver were assessed using immunoblot and quantitative analysis (n = 3 mice/ group). F, Liver tissues from each group processed for Masson's trichrome staining (n = 3 mice/ group, magnification, x400). G, Hydroxyproline of livers removed from olive‐induced and CCL4‐induced mice with or without TIM‐4 interference (n = 3 mice/ group). H, Inflammatory factors in livers were measured by enzyme‐linked immunosorbent assay (ELISA) (n = 3 mice/ group). ***P < .0001. Values represent the mean ± SD of at least three independent experiments

TIM‐4 was demonstrated to be present in KCs at high levels in CCL4‐induced liver fibrosis. To investigate whether TIM‐4 interference in KCs can reverse CCL4‐induced liver fibrosis, we administered TIM‐4 mAb (0.35 mg/mouse, once per week, for six weeks, red fluorescence labelled) via caudal vein injection to block the function of TIM‐4 in KCs. KCs were then isolated from CCL4‐induced mice. Red fluorescence was obviously observed in the TIM‐4 mAb injection group but was not observed in the control and NC group (Figure 2A), indicating that the function of TIM‐4 in KCs was successfully disrupted. Livers from control mice exhibited extensive destruction of liver structure, along with abnormal collagen deposition, whereas the collagen accumulation in the TIM‐4 mAb group was markedly reduced, with nearly normal liver architecture (Figure 2B). The histological findings were verified biochemically by hydroxyproline assay (Figure 2C).

It has been demonstrated that polymers greater than 175 nm in diameter cannot pass through the endothelial gap to make contact with hepatocytes but are mainly taken up by macrophages in the liver.^31^ We utilized mannose‐conjugated polymers (greater than 175 nm in diameter) to deliver TIM‐4 siRNA (TIM‐4‐siRNA) or its scrambled control siRNA in vivo. KCs were isolated from each group, and the expression of TIM‐4 in the KCs was found to be inhibited in the TIM‐4 interference (Figure 2D,2). TIM‐4 interference had no effect on the liver parenchyma in the olive‐induced mice. There was dense collagen deposition in the CCL4‐induced mice, whereas the TIM‐4 interference mice had a substantially normal structure (Figure 2F). The histological findings were verified biochemically by hydroxyproline assay (Figure 2G). In addition, CCL4‐induced mice had mild pro‐inflammatory response (IL‐1β and TNF‐α) in liver (Figure 2H). Chronic stimulation of anti‐inflammatory (IL‐4, IL‐10, IL‐13 and TGF‐β1) factors is related to liver fibrosis progression. Moreover, we found that among these TGF‐β1 expression was extremely high in CCL4‐induced mice, but was significantly reduced after TIM‐4 interference (Figure 2H). These data indicate that TIM‐4 activation in KCs is involved in the pathogenesis of CCL4‐injured liver fibrosis, and TIM‐4 interference contributes to liver fibrosis resolution.

### 3.3 TIM‐4 Interference of KCs reduces pro‐fibrotic factors required for HSC function

**Figure 3:**
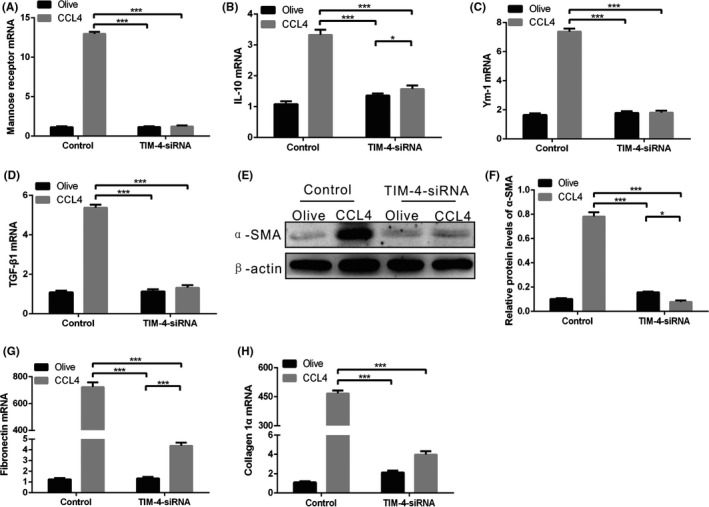
KCs from CCL4‐induced liver fibrosis have a pro‐fibrotic phenotype. A‐D, Mannose receptor, interleukin‐10 (IL‐10), Ym1 and transforming growth factor β1 (TGF‐β1) mRNAs in KCs were measured by quantitative PCR (n = 3 mice/ group). E, F, The α‐SMA expression of HSCs cultured in KCs supernatant from olive‐induced and CCL4‐induced mice with or without TIM‐4 interference was determined by immunoblot and quantitative analysis. G, H, Fibronectin and collagen 1α mRNAs in HSCs cultured in KC supernatant from olive‐induced and CCL4‐induced mice with or without TIM‐4 interference was measured by quantitative PCR. *P < .05; ***P < .0001. Values represent the mean ± SD of at least three independent experiments

Because TIM‐4 interference in KCs contributes to fibrosis resolution, we hypothesized that TIM‐4 interference would inhibit the polarization of KCs to a pro‐fibrotic phenotype. Mannose receptor, IL‐10 and Ym1 (pro‐fibrotic marker) mRNAs were significantly increased in CCL4‐induced mice (Figure 3A,B,C). In contrast, TIM‐4 interference mice had low mannose receptor, IL‐10 and Ym1 mRNA expression levels (Figure 3A,B,C). Furthermore, TGF‐β1 expression in KCs from TIM‐4 interference mice was similar to that in olive‐induced control mice (Figure 3D). TGF‐β1 induces the differentiation of HSCs into myofibroblast‐like cells; therefore, we isolated HSCs that were incubated with KCs from each group. The expression of α‐SMA was less in HSCs incubated with KCs from TIM‐4 interference mice than from CCL4‐induced control mice, indicating that KC‐derived TGF‐β1 is crucial for HSC differentiation (Figure 3E,F). In addition, HSCs incubated with KCs from CCL4‐induced control mice showed a 175‐fold increase in fibronectin mRNA and a 120‐fold increase collagen 1α mRNA compared to HSCs incubated with KCs from CCL4‐induced TIM‐4 interference mice (Figure 3G,H). Collectively, these data reveal that KCs from TIM‐4 interference mice exhibited an anti‐fibrotic effect by decreasing the pro‐fibrotic factors that are required for HSC function.

### 3.4 KC‐derived TGF‐β1 is required for liver fibrosis

**Figure 4:**
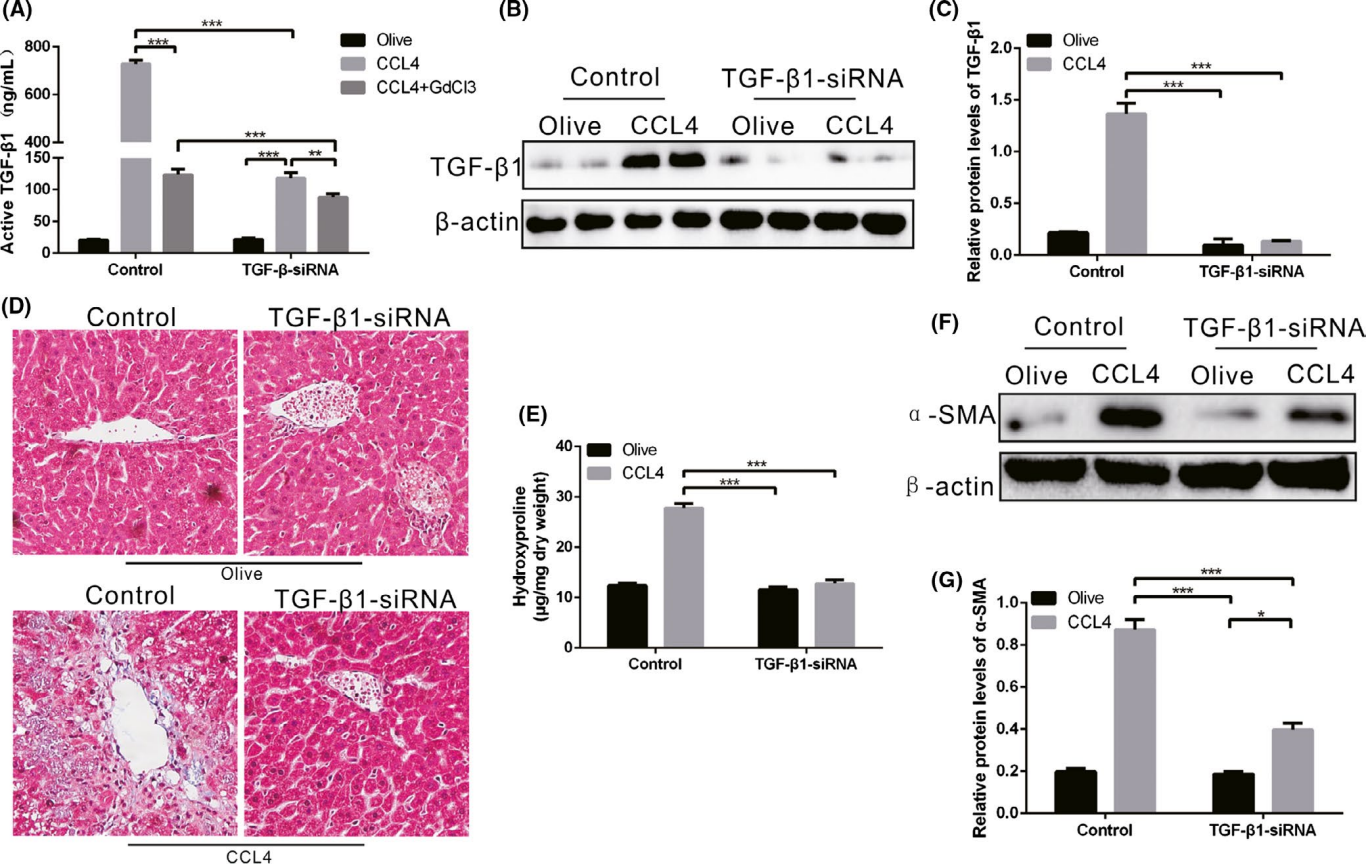
KC‐derived TGF‐β1 is required for liver fibrosis. A, Mannose‐conjugated polymers were used to deliver TGF‐β1 siRNA or its scrambled control siRNA in vivo. Additionally, GdCl3 was injected to block hepatic KCs function. The concentration of TGF‐β1 was measured by ELISA (n = 3 mice/ group). B‐C, The expression of TGF‐β1 in KCs from olive‐induced and CCL4‐induced mice with or without TGF‐β1 interference was determined by immunoblot and quantitative analysis. D, Liver tissues from each group were stained with Sirius red (n = 3 mice/ group, magnification, ×400). E, Hydroxyproline of livers removed from olive‐induced and CCL4‐induced mice with or without TGF‐β1 interference (n = 3 mice/ group). F‐G, The α‐SMA expression of HSCs cultured in KC supernatant from olive‐induced and CCL4‐induced mice with or without TGF‐β1 interference was determined by immunoblot and quantitative analysis. *P < .05; **P < .001; ***P < .0001. Values represent the mean ± SD of at least three independent experiments

We surmised that the above observations of HSC differentiation resulted from the reduction of KC‐derived TGF‐β1 in CCL4‐induced TIM‐4 interference mice. To determine whether KC‐derived TGF‐β1 mediated these change, we utilized mannose‐conjugated polymers to deliver TGF‐β1 siRNA (TGF‐β1‐siRNA) or its scrambled control siRNA in vivo. Additionally, we injected GdCl3 (10 mg/kg) via the caudal vein prior to CCL4 treatment to block hepatic KC function. Liver homogenates from CCL4‐induced control mice had a high concentration of TGF‐β1, while a decreased concentration of TGF‐β1 was observed in CCL4‐induced control mice with GdCl3 treatment (Figure 4A). Moreover, after TGF‐β1 siRNA treatment, the CCL4‐induced mice with GdCl3 treatment had a lower concentration of TGF‐β1 than that in CCL4‐induced mice without GdCl3 treatment, suggesting that TGF‐β1 secretion is mainly derived from KCs during fibrosis progression (Figure 4A). KCs were then isolated from each group, and the expression of TGF‐β1 was inhibited in KCs that had undergone TGF‐β1 interference (Figure 4B,C). Meanwhile, TGF‐β1 interference mice had substantially normal livers (Figure 4D). The histological findings were verified biochemically by hydroxyproline assay (Figure 4E). The expression of α‐SMA was lower in HSCs incubated with KCs from the TGF‐β1 interference mice compared with CCL4‐induced control mice (Figure 4F). Taken together, KC‐derived TGF‐β1 expression regulates the conversion of a fibrotic phenotype in vivo.

### 3.5 TIM‐4 interference of KCS decreases Mitophagy VIA Akt1 IN liver fibrosis

**Figure 5:**
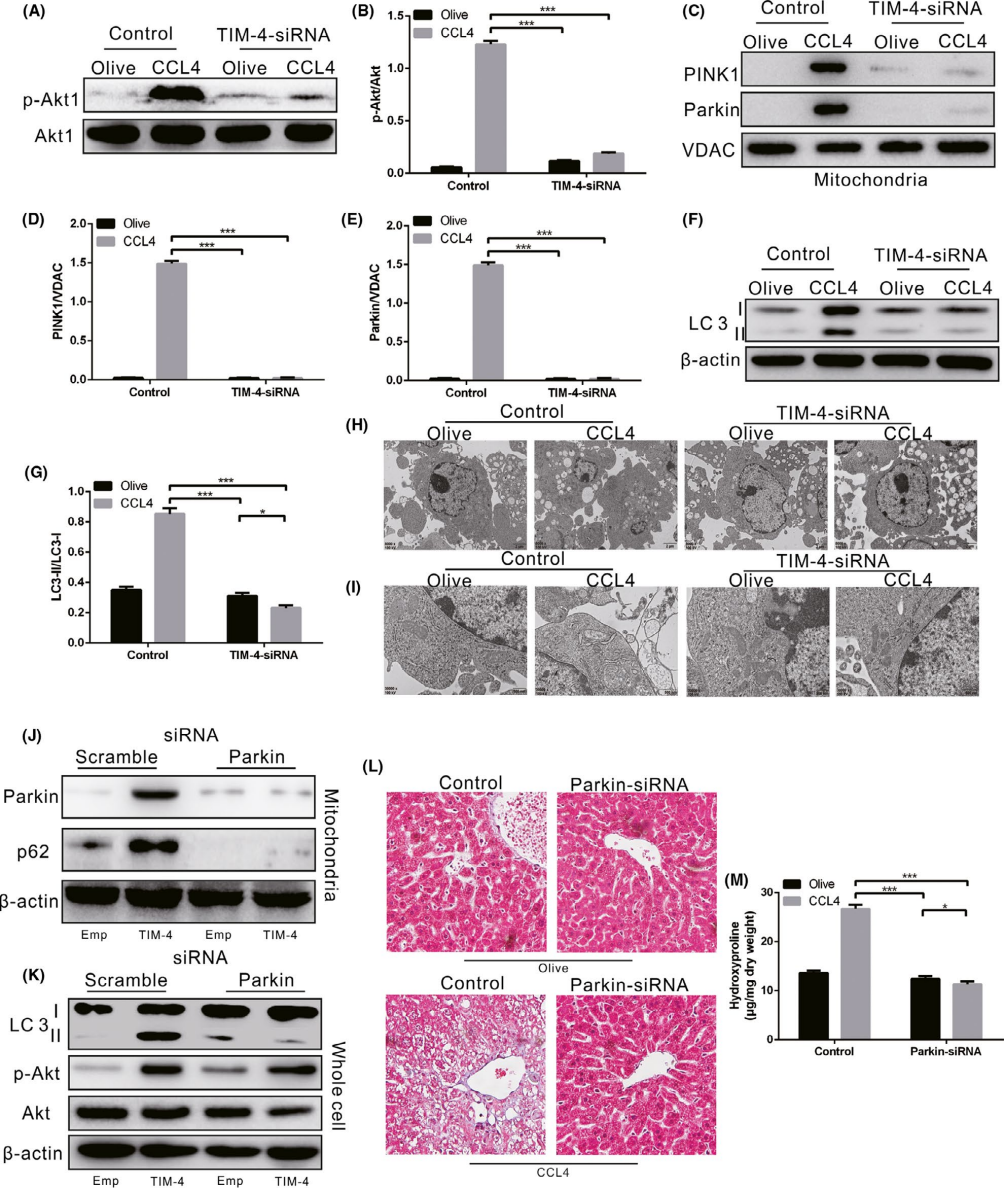
TIM‐4 in KCs mediates mitophagy via Akt1 during liver fibrosis. A‐B, The expression of p‐Akt1 in KCs from olive‐induced and CCL4‐induced mice with or without TIM‐4 interference was determined by immunoblot and quantitative analysis (n = 3 mice/ group). C‐G, The expression levels of PINK1, Parkin and LC3 II/I in KCs from olive‐induced and CCL4‐induced mice with or without TIM‐4 interference were determined by immunoblot and quantitative analysis (n = 3 mice/ group). H, I, Vacuoles (Scale bars: 2 μm) and mitochondria (Scale bars: 500 nm) in KCs from olive‐induced and CCL4‐induced mice with or without TIM‐4 interference were observed under transmission electron microscopy (n = 3 mice/ group). J‐K, The expression levels of mitochondrial Parkin and p62, along with LC3 II/I, were measured in KCs transfected with scrambled or Parkin2 siRNA in combination with empty or TIM‐4 overexpression adenovirus (n = 3 mice/ group). L, Liver tissues from olive‐induced and CCL4‐induced mice with or without Parkin interference were stained with Sirius red (n = 3 mice/ group, magnification, x400). M, Hydroxyproline of livers removed from olive‐induced and CCL4‐induced mice with or without Parkin interference (n = 3 mice/ group). *P < .05; ***P < .0001. Values represent the mean ± SD of at least three independent experiments

Akt1 is involved in various diseases and can be activated by many endogenous and exogenous insults; therefore, we next determined the expression of Akt1 in each group. The p‐Akt1 expression in CCL4‐induced control mice was elevated, but KCs from CCL4‐induced TIM‐4 interference mice showed low expression of p‐Akt1 (Figure 5A,B), which indicated that Akt activation is regulated by TIM‐4 during fibrosis.

It has been demonstrated that macrophage autophagy is an anti‐inflammatory and anti‐fibrogenic pathway, and macrophages in mice with ATG5 deficiency induce exacerbated hepatic inflammation and fibrosis.^12^ However, the contribution of mitophagy in KCs to liver fibrosis is limited. We discovered that mitochondria isolated from CCL4‐induced control mice had greater PINK1, Parkin and LC3‐II/I expression levels compared to olive‐induced mice (Figure 5C,D,E,F,G), suggesting that mitophagy occurred as indicated by autophagosome formation. In contrast, the TIM‐4 interference mice showed extensive inhibition of PINK1, Parkin and LC3‐II/I expression (Figure 5C,D,E,F,G).

To visually explore the effect of TIM‐4 interference on KCs, we applied electron microscopy to examine KC mitophagy. KCs isolated from CCL4‐induced control mice had an increased presence of vacuoles compared to KCs isolated from olive‐induced control mice. Conversely, KCs from TIM‐4 interference mice had no visible vacuoles (Figure 5H). KCs isolated from CCL4‐induced control mice showed irregularly shaped mitochondria with disorganized cristae, while KCs from TIM‐4 interference mice had normal mitochondria (Figure 5I).

Next, we utilized mannose‐conjugated polymers to deliver Parkin siRNA (Parkin siRNA) or its scrambled control siRNA in vivo. KCs were isolated from each group and then treated with TIM‐4 overexpression adenovirus or empty adenovirus in vitro. The TIM‐4 overexpression and Parkin interference KCs showed very low or no Parkin, p62 and LC3‐II/I expression (Figure 5J,K). p‐Akt1 expression in Parkin interference KCs was markedly suppressed, but was increased in Parkin interference KCs with TIM‐4 overexpression (Figure 5J, K), providing evidence that the activation of Akt1 in KCs induces mitophagy. Dense collagen deposition was observed in CCL4‐induced mice, but the Parkin interference mice had normal liver architecture (Figure 5L). These results were confirmed by hydroxyproline analysis (Figure 5M).

### 3.6 TIM‐4 mediates mitochondrial ROS VIA Akt1 to induce KC Mitophagy

**Figure 6:**
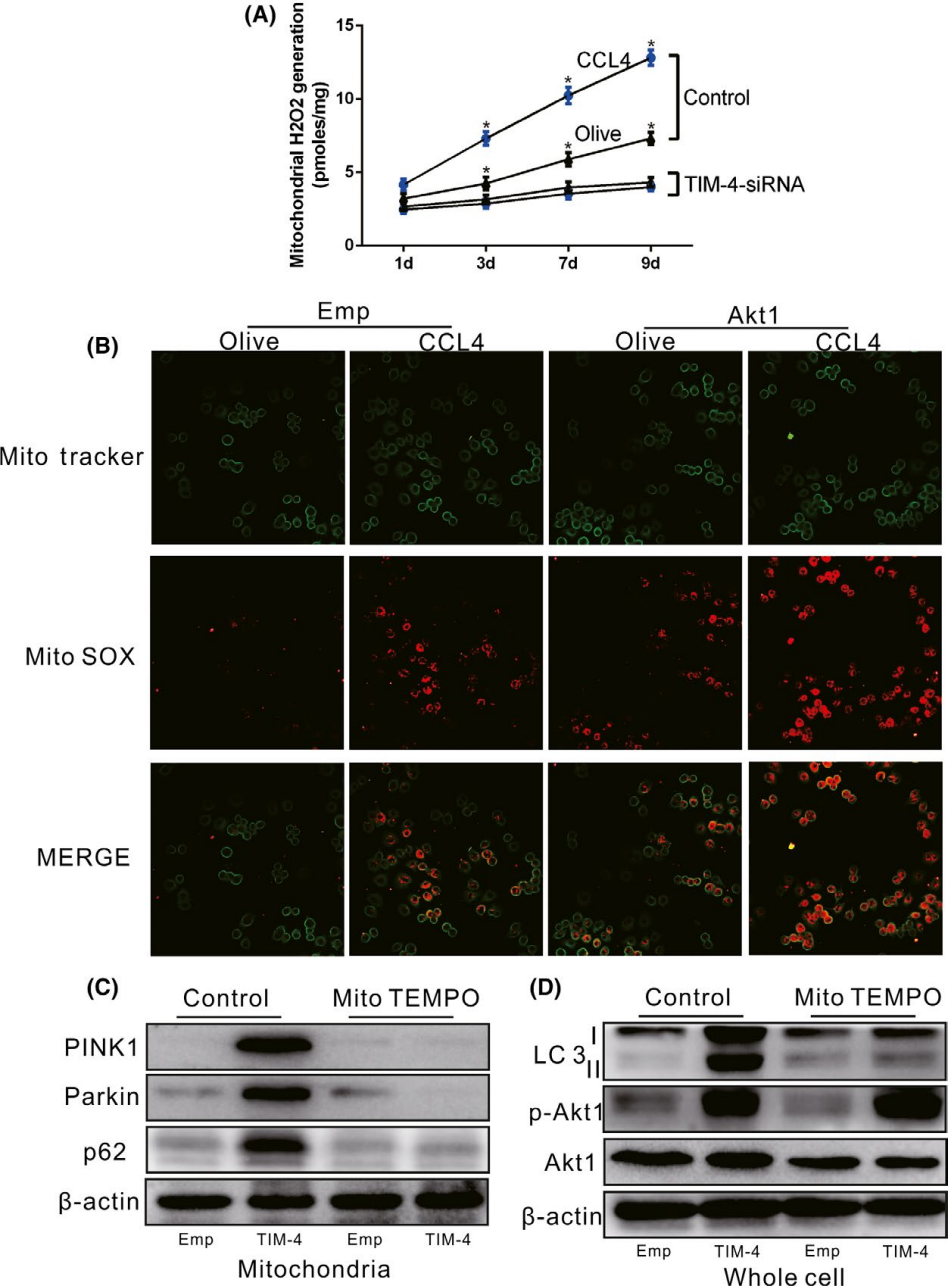
TIM‐4 mediates mitochondrial ROS via Akt1 to induce KC mitophagy during liver fibrosis. A, Mitochondria were isolated from KCs, and H_2_O_2_ production was measured (n = 3 mice/ group). B, Representative fluorescence images of KCs transfected with empty or Akt1 overexpression adenovirus from olive‐ or CCL4‐treated mice. KCs were co‐stained with MitoTracker and MitoSOX (n = 3 mice/ group, Scale bars: 50 μm). C, D, Mitochondrial Parkin and p62, and LC3 II/I expression levels were measured in KCs treated with vehicle or MitoTEMPO in combination with empty or TIM‐4 overexpression adenovirus (n = 3 mice/ group). *P < .05. Values represent the mean ± SD of at least three independent experiments

Evidence has shown that mitophagy is induced by ROS and that Akt1 increases mitochondrial ROS.^32^, ^33^ We showed that KC mitochondria isolated from CCL4‐induced mice produced significantly more H2O2 compared to those from the olive control. In contrast, KC mitochondria isolated from TIM‐4 interference mice had significantly reduced H2O2 production compared to the control (Figure 6A). To verify that Akt1 modulates mitochondrial ROS, KCs were co‐stained with MitoSOX red and MitoTracker green to observe ROS production by immunofluorescence. The results showed that after CCL4 treatment, KCs had increased MitoSOX fluorescence and was colocalized with MitoTracker (Figure 6B). Moreover, the overexpression of Akt1 in KCs with CCL4 treatment showed further enhanced MitoSOX fluorescence intensity (Figure 6B). To confirm that ROS production induced mitophagy via TIM‐4, immunoblot analysis was applied and showed that mitophagy in KCs overexpressing TIM‐4 was distinctly inhibited by mitochondrial‐targeted antioxidant (MitoTEMPO) (Figure 6C,D). However, MitoTEMPO treatment had no effect on Akt1 activation in KCs in the presence of TIM‐4 overexpression (Figure 6C,D). These findings suggest that TIM‐4 mediates mitochondrial H2O2 via Akt1 to regulate KC mitophagy during fibrosis.

### 3.7 KC Mitophagy is required for TGF‐β1 expression in liver fibrosis

**Figure 7:**
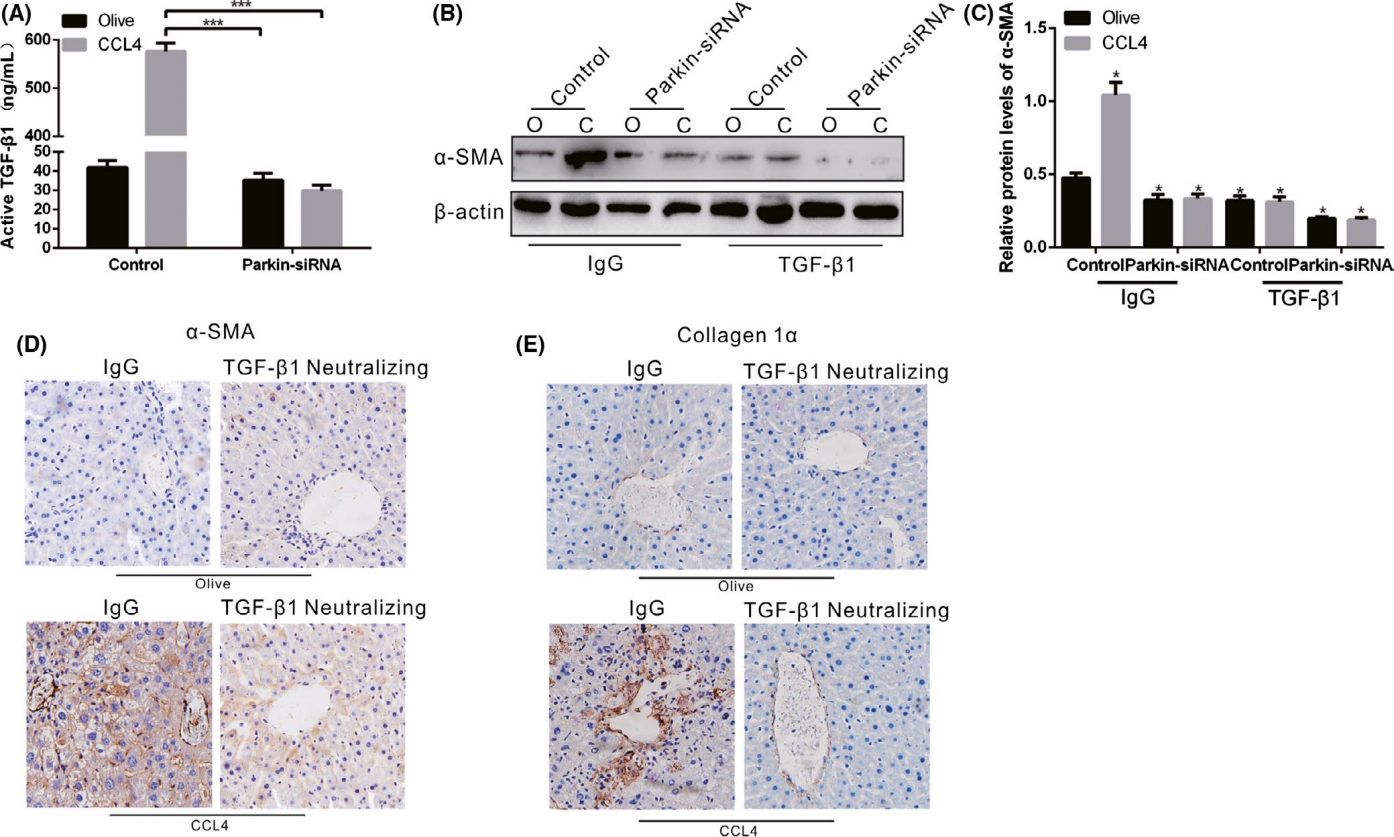
KC mitophagy regulates TGF‐β1 during liver fibrosis. A, The concentration of TGF‐β1 in livers from olive‐induced and CCL4‐induced mice with or without Parkin interference was measured by ELISA (n = 3 mice/ group). B, C, The α‐SMA expression in HSCs cultured in KC supernatant from olive‐induced and CCL4‐induced mice with or without Parkin interference was determined by immunoblot and quantitative analysis. The supernatant was pre‐incubated with IgG control or neutralized with TGF‐β1 antibody (10 mg/mL). D, E, The expression levels of α‐SMA and collagen 1α in olive‐induced and CCL4‐induced mice with IgG or TGF‐β1 antibody treatment were assessed using immunohistochemistry (n = 3 mice/ group, magnification, x400). *P < .05; ***P < .0001. Values represent the mean ± SD of at least three independent experiments

To explore whether KC mitophagy is required for TGF‐β expression, we utilized mannose‐conjugated polymers to deliver Parkin siRNA (Parkin siRNA) or its scrambled control siRNA in vivo. Liver homogenates from CCL4‐induced control mice had a high concentration of TGF‐β1, whereas a decreased concentration of TGF‐β1 was observed in CCL4‐induced Parkin interference mice (Figure 7A). Because mitophagy regulates KC‐derived TGF‐β1 can induce HSC differentiation and function, but whether KC‐derived TGF‐β1 was the responsible factor for HSC differentiation was unclear. By using a TGF‐β1 neutralizing antibody, HSCs incubated with neutralized KC supernatant failed to differentiate, shown by markedly lower α‐SMA expression compared to the olive control (Figure 7B,C). Moreover, immunohistochemical analysis showed that the expression of α‐SMA and collagen 1α in CCL4‐induced mice without TGF‐β1 neutralization was prominently increased, but these were decreased in CCL4‐induced mice with TGF‐β1 neutralization (Figure 7D,E). Overall, it was verified that TIM‐4‐induced mitophagy in KCs is required for KC‐derived TGF‐β1 expression, which in turn promotes fibrosis development.

### 3.8 TIM‐4 interference of KCS increases the development of fibrosis after liver transplantation

**Figure 8:**
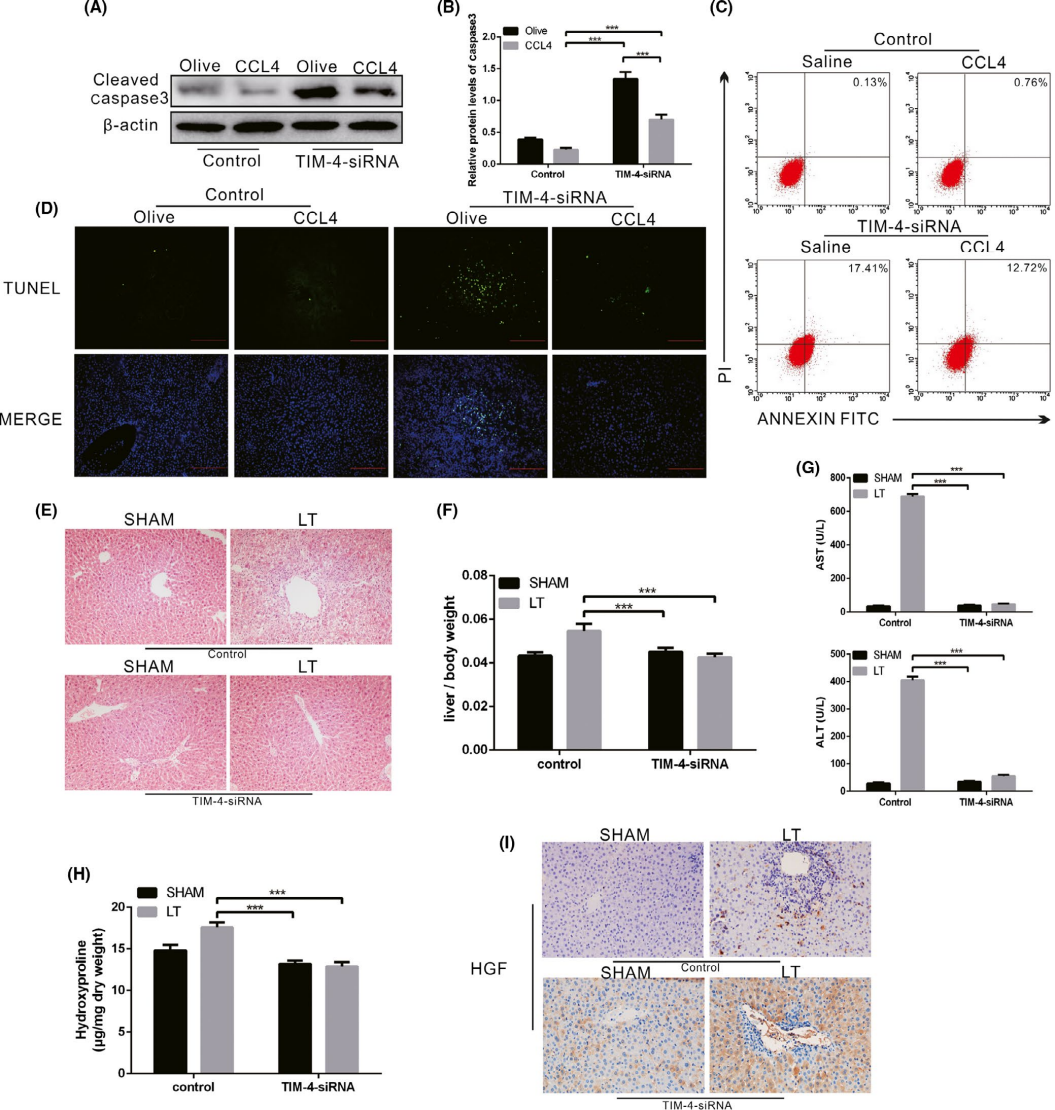
TIM‐4 in KCs mediates mitophagy via Akt1 during liver fibrosis. A, B, The expression levels of cleaved caspase‐3 in KCs from olive‐induced and CCL4‐induced mice with or without TIM‐4 interference were determined by immunoblot and quantitative analysis (n = 3 mice/ group). C, The number of apoptotic KCs from olive‐induced and CCL4‐induced mice with or without TIM‐4 interference was determined with annexin V/PI staining and flow cytometric analysis (n = 3 mice/ group). D, Representative liver sections stained with terminal deoxynucleotidyl transferase‐mediated dUTP‐biotin nick end labelling assay (TUNEL) in sham and liver transplantation mice with or without TIM‐4 interference (n = 3 mice/ group). E, Representative micrographs of the pathological damage observed in sham and liver transplantation mice with or without TIM‐4 interference following staining with haematoxylin and eosin (n = 3 mice/ group, magnification, x400). F, The ratio of liver weight to body weight was measured in sham and liver transplantation mice with or without TIM‐4 interference (n = 5 mice/ group). G, Serum concentrations of alanine aminotransferase (ALT) and aspartate transaminase (AST) in sham and liver transplantation mice with or without TIM‐4 interference (n = 5 mice/ group). H, Hydroxyproline of livers removed from sham and liver transplantation mice with or without TIM‐4 interference (n = 5 mice/ group). I, The expression levels of hepatocyte growth factor (HGF) in sham and liver transplantation mice with or without TIM‐4 interference were assessed using immunohistochemistry (n = 5 mice/ group, magnification, x400). ***P < .0001. Values represent the mean ± SD of at least three independent experiments

A previous study has indicated that macrophages typically exhibit apoptosis resistance in chronic disease.^34^ We determined that the expression of caspase‐3 in KCs isolated from CCL4‐induced control mice was extremely low, whereas TIM‐4 interference of KCs resulted in an increase of caspase‐3 expression (Figure 8A,8). KCs and liver tissues from CCL4‐induced control mice showed apoptotic resistance, but TIM‐4 interference counteracted the effect of resistant apoptosis to some extent (Figure 8C,D).

It is established that KCs play a critical role in the immunological balance associated with fibrosis after liver transplantation.^35^ To investigate whether TIM‐4 interference of KCs functioned in fibrosis after liver transplantation, we established orthotopic liver transplant models. Liver tissues from LT mice exhibited collagen proliferation in the portal area and central venous area, as well as and hepatocyte necrosis or steatosis, which were characterized by early cirrhosis (Figure 8E). However, liver tissue from TIM‐4 interference mice had normal architecture (Figure 8F). The histological findings were verified biochemically by hydroxyproline assay (Figure 8H). In addition, the ratio of liver weight to body weight was significantly reduced in the TIM‐4 interference mice compared to the LT control mice (Figure 8F). Furthermore, the liver function of mice with TIM‐4 interference was visibly improved (Figure 8G). Immunohistochemical analysis showed that TIM‐4 interference mice with LT had increased hepatocyte growth factor (HGF) expression (Figure 8I). These results indicate that TIM‐4 interference in KCs may potentially improve the development of fibrosis after liver transplantation.

## 4 DISCUSSION

Due to its increasing prevalence and substantial clinical consequences, liver fibrosis has received much attention. Irrespective of the underlying aetiologies, fibrosis will progress to cirrhosis and liver cancer.^2^, ^36^ Tremendous advances have been made in understanding the related molecular mechanisms and in developing efficacious therapeutics for liver fibrosis.^37^ In recent years, the treatment of liver fibrosis has progressed from anti‐inflammatory treatment to multi‐modal treatment targeting HSCs to prevent the formation of collagen fibres and promote collagen degradation. However, there are few studies targeting macrophages for liver fibrosis treatment. It has been demonstrated that macrophage conditional depletion could attenuate models of liver and lung injury in vivo.^11^ Thus, KC functionally act on the pathogenesis of liver fibrosis.

TIM‐4 is mainly expressed in APCs. KCs are the largest APCs in vivo, and they selectively express TIM‐4.^11^ Recent data have shown that TIM‐4 blockade inhibits the production of pro‐inflammatory cytokines but does not affect the phagocytosis of apoptotic bodies mediated by DCs.^38^ Furthermore, the disruption of TIM‐4 signalling inhibits macrophage and neutrophil migration/function and suppresses the activation of TLR2/4/9‐dependent signalling. Therefore, TIM‐4 plays an important role in regulating the immune function of macrophages,^22^ but there is currently no evidence of TIM‐4 functioning in liver fibrosis. In the present study, we found that CCL4‐induced liver fibrosis mice showed KCs were predominant number of macrophages in liver (>90%) with high levels of TIM‐4 expression instead of other liver non‐parenchymal cells. Therefore, KC‐expressed high TIM‐4 concentrations may be associated with the progression of liver fibrosis. Based on previous data, we utilized TIM‐4 mAb or TIM‐4‐siRNA in vivo for investigating the association between TIM‐4 and liver fibrosis. It was verified that TIM‐4 interference could essentially decrease the hydroxyproline and collagen deposition in CCL4‐induced liver fibrosis.

Early stage of liver fibrosis is related to inflammatory condition, but it will soon be in immunosuppression due to different macrophage polarization during liver fibrosis.^11^ Macrophages have bidirectional role in the formation of liver fibrosis, which are divided into two types, type I and type II. Type II macrophage is regulated by Th2 factor to produce IL‐10 and TGF‐β, and is associated with allergy, apoptosis and fibrosis.^39^ It is well established that TGF‐β1 is one of the main factors in the induction of fibrosis.^39^ It can promote the aggregation of fibroblasts and inflammatory cells, as well as the synthesis of collagen and fibrin, which further results in extracellular matrix deposition and degradation.^40^ The content of TGF‐β1 in normal liver cells is extremely low to none. KCs synthesize and secrete large amounts of TGF‐β1 in various factors, leading to hepatocyte injury.^41^ Our data demonstrated that CCL4‐induced mice had mild pro‐inflammatory response, and anti‐inflammatory factor TGF‐β1 was extremely high in CCL4‐induced mice, but was significantly reduced after TIM‐4 interference, so we focused on the relation between TIM‐4 and TGF‐β1. Meanwhile, KCs from TIM‐4 interference mice had decreased levels of pro‐fibrotic markers (mannose receptor, IL‐10, Ym1 and TGF‐β). Moreover, HSCs incubated with KCs from TIM‐4 interference mice showed low expression levels of fibronectin and collagen 1α, which are required for HSC function. The administration of GdCL3 (a functional blocking reagent of KCs) in CCL4‐induced mice obviously resulted in a low concentration of TGF‐β1, which indicates that KCs are the primary source of TGF‐β1 during fibrosis progression. Meanwhile, TGF‐β1 interference in KCs decreased HSC activation and contributed to liver fibrosis resolution.

Mitophagy is a cell survival mechanism that is increased under stress conditions in an attempt to retain cell homeostasis. Mitophagy has been implicated in many physiological processes, such as tumorigenesis, neurological and immunological disorders.^42^, ^43^ It has been demonstrated that mitochondrial dysfunction is accompanied by a large amount of ROS production, mitochondrial division/fusion changes, and there is impaired mitophagy in the early stage of Alzheimer's disease.^44^ Mice with Parkin and PINK1 genetic deletion had symptoms similar to Parkinson's disease, with progressive mitochondrial damage.^45^ Thus, Parkin and PINK1 play key roles in the process of clearing damaged mitochondria. Akt1, a pro‐survival kinase, is known to mediate mitochondrial H2O2 generation. Akt1 activation is strongly associated with regulating survival and many fibrotic remodelling processes.^46^ The phosphorylation level of Akt is associated with HSC proliferation and collagen I expression.^47^ Moreover, TGF‐β1 regulates Akt activation in myofibroblasts, and the inhibition of Akt diminishes TGF‐β1‐induced fibrosis.^46^, ^47^ In human lung fibroblasts, TGF‐β1 has been shown to decrease autophagy by activating the Akt pathway.^48^ Our results showed that KCs from CCL4‐induced mice had increased ROS production, mitophagy activation and TGF‐β1 secretion. The KC mitochondria were observed with an irregular shape and disorganized cristae during liver fibrosis. To clarify the pathway, we overexpressed and blocked all aspects of the mentioned pathway and demonstrated that TIM‐4 interference in KCs inhibited Akt1‐mediated ROS production, resulting in the suppression of PINK1, Parkin and LC3‐II/I activation and the reduction of TGF‐β1 secretion during liver fibrosis.

Macrophages generally exhibit apoptotic resistance in chronic disease, and their long‐term survival is related to disease progression.^49^ We found that liver fibrosis mice had apoptotic resistance, but TIM‐4 interference counteracted apoptotic resistance to some extent. Liver transplantation is regarded as an important method for treating end‐stage liver disease. However, as time increases after the transplantation, liver fibrosis becomes one of the main causes of liver failure. After transplantation, livers will gradually progress into fibrosis due to ischaemia‐reperfusion injury and immune environment changes.^50^ The intricate mechanisms involve in multiple factors^51^, ^52^: The metabolic rate of the donor liver is reduced during cold ischaemia, and the stromal cells such as KCs are damaged, activating NF‐κB pathway to express various inflammatory mediators such as adhesion factors and chemokines following stress reaction occur after thawing. The application of immunosuppressive agents inhibits lymphocyte function and changes the proportion of lymphocyte subpopulations. The balance of Th1/Th2 cell ratio is biased towards immunosuppressive Th2 differentiation, which contributes to the formation of immune tolerance and promotes fibrosis. Studies have reported that 85.5% of patients with normal liver function have various degrees of fibrotic changes.^52^ In the current study, liver transplant models were conducted. We demonstrated that the livers after LT were characterized by early cirrhosis, whereas TIM‐4 interference mice had normal liver architecture and function. Additionally, TIM‐4 interference mice had a decreased liver/body weight ratio and increased HGF expression, indicating that TIM‐4 interference potentially attenuates the development of fibrosis after LT.

**Figure 9:**
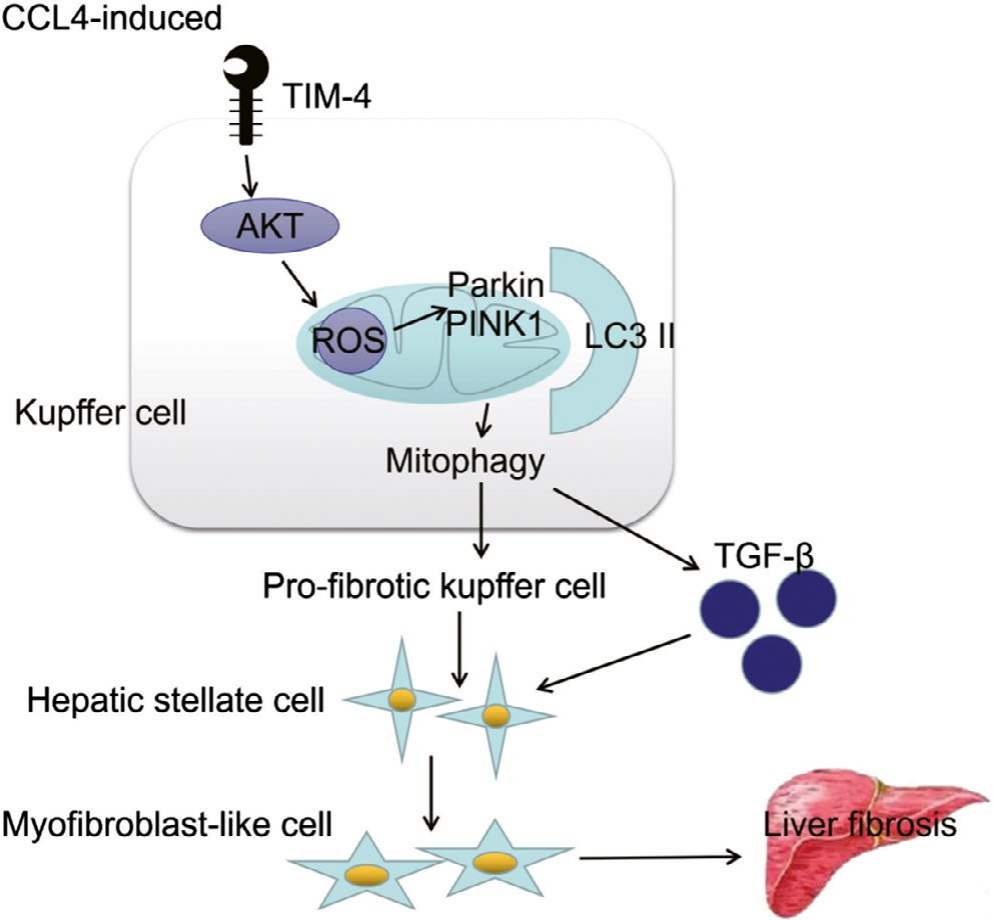
TIM‐4 mediates mitochondrial ROS via Akt1 to regulate mitophagy in KCs, which are associated with fibrosis development

As summarized in Figure 9, our study clarified that TIM‐4 mediated Akt1/mitophagy pathway in KC is linked to pro‐fibrotic polarization, apoptotic resistance and is required for fibrosis development. TIM‐4 interference in KCs as a novel immune target may be considered as a promising anti‐fibrogenic to treat liver fibrosis progression.

## COMPETING INTERESTS

All of the authors declare that there are no competing interests.

## AUTHOR CONTRIBUTIONS

WH and YFC participated in data analysis. WH and CGY participated in research design. WH participated in writing of the paper. WH, CGY, WJY, and DMH participated in performance of the research. YFC and GJP participated in critical revision of the paper. YFC and GJP participated in approval of paper.

## Data Availability

The datasets used and/or analysed in the current study are available from the corresponding author on reasonable request.
