# Supplementary figures and images for: TIM‐4 interference in Kupffer cells against CCL4‐induced liver fibrosis by mediating Akt1/Mitophagy signalling pathway
Source: Cell Prolif. 2019 Nov 22;53(1):e12731. doi: 10.1111/cpr.12731 (PMC6985653; doi:10.1111/cpr.12731)

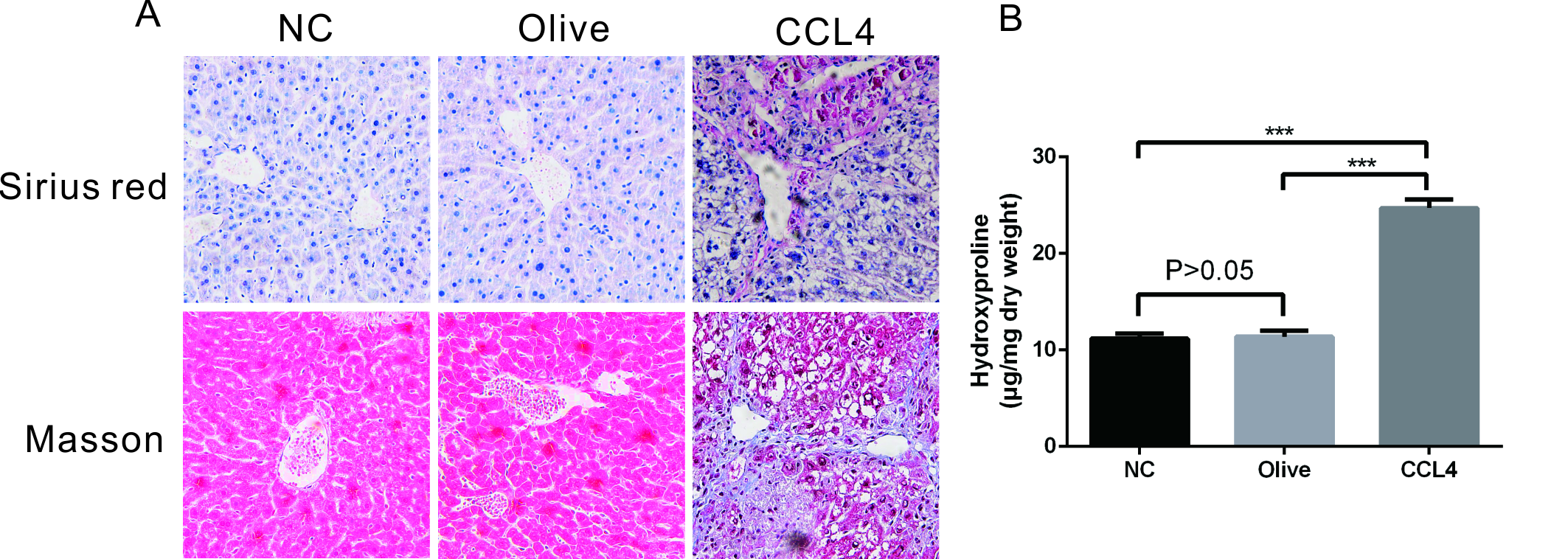

Supplement: Supplementary file 1 [file CPR-53-e12731-s001.tif]

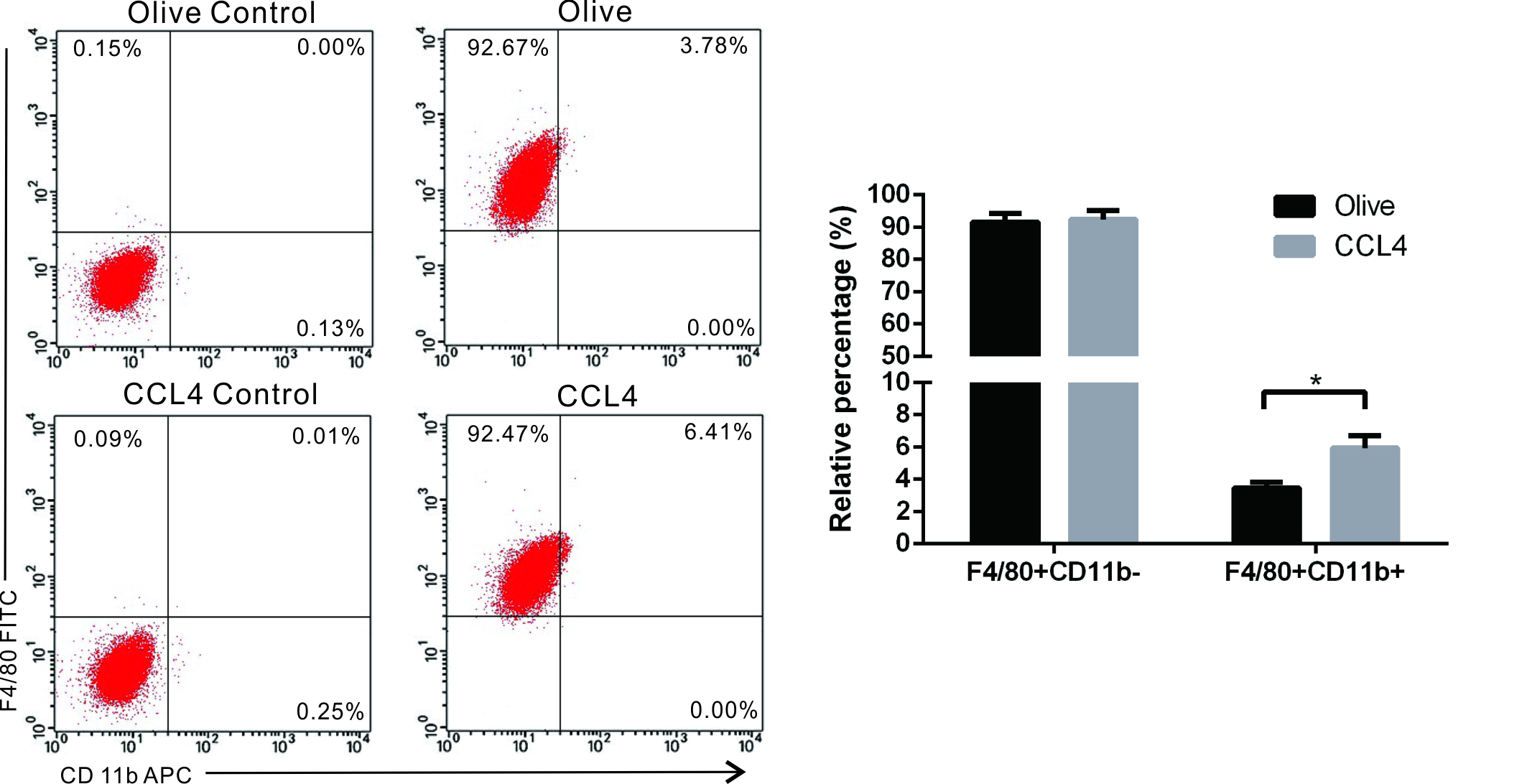

Supplement: Supplementary file 2 [file CPR-53-e12731-s002.tif]
