# Supplementary material for: TIM‐4 interference in Kupffer cells against CCL4‐induced liver fibrosis by mediating Akt1/Mitophagy signalling pathway
Source: Cell Prolif. 2019 Nov 22;53(1):e12731. doi: 10.1111/cpr.12731 (PMC6985653; doi:10.1111/cpr.12731)
Supplement: Supplementary file 3 [file CPR-53-e12731-s003.doc]

**FIGURE S1** (A) The liver fibrosis models were established with CCL4 treatment. Olive- induced mice were injected with commensurable olive. NC (Negative control) as a blank control. Liver tissues from each group were processed with sirius red and masson’s trichrome staining (n=3 mice/ group, magnification, x400). (B) Hydroxyproline of livers removed from each group (n=3 mice/ group). ***P < 0.0001. Values represent the mean ± SD of at least three independent experiments.

**FIGURE S2** (A) The macrophages extracted from olive and CCL4 induced models’ livers were identified with F4/80 and CD11b by flow cytometry (n= 3 mice/ group). Control, F4/80 and CD11b homologous IgG was used to exclude non-specific staining in the extracted cells. F4/80+ cells were macrophages, F4/80+CD11b+ cells were macrophages from circulation, F4/80+CD11b- cells were KCs. (B) The quantitative analysis of flow cytometry. *P < 0.05. Values represent the mean ± SD of at least three independent experiments.
